# Successful Management of Occult Breast Cancer with a Background of Anti-Melanoma Differentiation-Associated Gene 5 Antibody-Positive Interstitial Pneumonia: A Case Report

**DOI:** 10.70352/scrj.cr.25-0050

**Published:** 2025-05-08

**Authors:** Mikako Ishii, Yoshiya Horimoto, Yoichi Koyama, Kayo Adachi, Ai Ueda, Takahiko Kawate, Hiroshi Kaise, Kimito Yamada, Eiichi Sato, Shinji Abe, Takashi Ishikawa

**Affiliations:** 1 Department of Breast Surgical Oncology, Tokyo Medical University, Tokyo, Japan; 2 Department of Breast Oncology, Ibaraki Medical Center, Tokyo Medical University, Ami, Ibaraki, Japan; 3 Department of Breast Oncology, Hachioji Medical Center, Tokyo Medical University, Hachioji, Tokyo, Japan; 4 Department of Pathology (Medical Research Center), Institute of Medical Science, Tokyo Medical University, Tokyo, Japan; 5 Department of Respiratory Medicine, Tokyo Medical University, Tokyo, Japan

**Keywords:** anti-MDA5 antibody-positive associated interstitial pneumonia, occult breast cancer, chemotherapy, dermatomyositis

## Abstract

**INTRODUCTION:**

Anti-MDA5 (melanoma differentiation-associated gene 5) antibody-positive dermatomyositis is a severe subtype of dermatomyositis associated with rapidly progressive interstitial lung disease, which carries an extremely high mortality rate. Prompt diagnosis and therapeutic intervention are crucial for survival. Here, we report a rare case of occult breast cancer in a patient with anti-MDA5 antibody-positive associated interstitial pneumonia. Following the control of the lung disease with immunosuppressive therapy, the patient successfully underwent neoadjuvant chemotherapy (NAC) and curative surgery.

**CASE PRESENTATION:**

A 63-year-old woman presented with progressive dyspnea. Imaging tests revealed diffuse ground-glass opacities in both lungs and enlarged left axillary lymph nodes. Blood tests showed elevated KL-6 levels and anti-MDA5 antibodies. Although no skin lesions or myositis were observed, she was diagnosed with anti-MDA5 antibody-positive associated interstitial pneumonia. Immunosuppressive therapy, including steroid pulse therapy, tacrolimus, cyclophosphamide pulse therapy, and plasma exchange, was initiated, leading to an improvement in her lung condition. She was then initially referred to the department of plastic surgery for further evaluation of the enlarged left axillary lymph node. Excisional biopsy of the enlarged left axillary lymph node revealed triple-negative occult breast cancer (cTXN1M0, Stage IIA). After the patient was referred to our department, NAC was initiated, achieving a clinical partial response while avoiding exacerbation of the interstitial pneumonia. After completing NAC, a left axillary lymph node dissection was performed, and the final pathological diagnosis was ypTXN2aM0 (Stage IIIA). Postoperative radiotherapy was omitted due to the risk of worsening the interstitial lung disease, and capecitabine was administered for 6 months. The patient has remained recurrence-free for 3 years following treatment.

**CONCLUSIONS:**

This case highlights the successful management of triple-negative breast cancer under the constraints of anti-MDA5 antibody-positive associated interstitial pneumonia. To ensure the smooth implementation of breast cancer treatment while controlling interstitial pneumonia, close collaboration with respiratory physicians was essential for a successful outcome.

## Abbreviations

IVCY
: cyclophosphamide pulse therapy
MDA5
: melanoma differentiation-associated gene 5
NAC
: neoadjuvant chemotherapy
PE
: plasma exchange
TN
: triple-negative

## INTRODUCTION

Dermatomyositis is an autoimmune disease that mainly affects the muscles and skin, but it encompasses a variety of conditions, including classical dermatomyositis with typical skin symptoms and muscle weakness, as well as malignancy-associated dermatomyositis. Anti-MDA5 antibodies are one of the autoantibodies specific to dermatomyositis, discovered by Japanese researchers,^1)^ and are known to be positive in around 20% of dermatomyositis cases.^2–6)^ Anti-MDA5 antibody-positive dermatomyositis is known to be associated with a high frequency of rapidly progressive interstitial pneumonia, with few cutaneous and muscular symptoms, and has the poorest prognosis among interstitial pneumonia associated with dermatomyositis. Once the patient is in respiratory failure requiring oxygenation, the survival rate is extremely low, and therapeutic intervention is required as soon as possible after diagnosis.^7)^

Herein, we report a case of occult breast cancer associated with anti-MDA5 antibody-positive associated interstitial pneumonia, in which NAC was successfully completed and radical surgery was performed while treating the pneumonia.

## CASE PRESENTATION

**Fig. 1:**
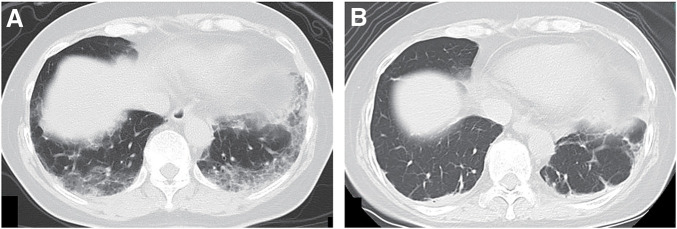
Chest CT scan findings. (**A**) Chest CT scan findings prior to initiation of treatment are shown (lung field striations). A diffuse, dorsal-dominant ground-glass opacity in the bilateral lung are seen. (**B**) Chest CT scan findings 6 weeks after starting treatment for anti-MDA5 antibody-positive interstitial pneumonia. MDA5, melanoma differentiation-associated gene 5

**Fig. 2:**
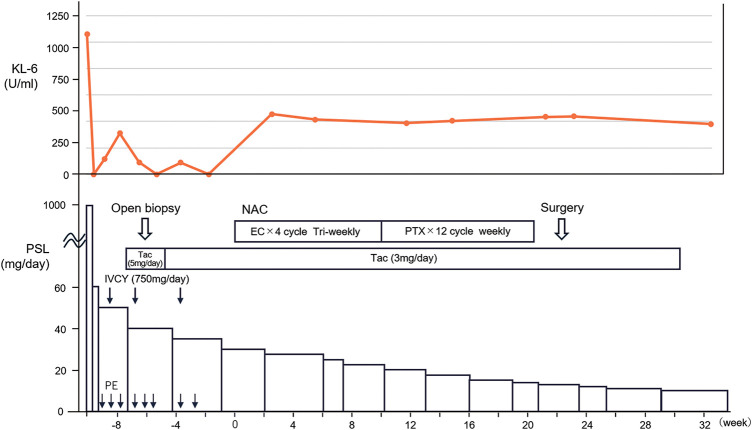
Schema of treatment progression. Clinical course with treatment details is shown. Orange curve indicates KL-6.

EC, epirubicin and cyclophosphamide; IVCY, cyclophosphamide pulse therapy; NAC, neoadjuvant chemotherapy; PE, plasma exchange; PSL, prednisolone; PTX, paclitaxel; Tac, tacrolimus

The patient was a 63-year-old woman. She experienced progressive dyspnea over several months, which eventually led her to seek hospital care. She had no medical history and no oral medications or supplements. Her family history included a daughter with systemic lupus erythematosus. Chest CT scan showed a diffuse, dorsal-dominant ground-glass opacity in the lungs and left axillary lymphadenopathy (**Fig. 1A**). Blood test results were positive for antinuclear antibody, but showed no increase in ANCA or IgG levels. Accordingly, a diagnosis of interstitial pneumonia was made, and steroid pulse therapy (methylprednisolone succinate 1 g × 3 days) was initiated, with the dose being reduced to 60 mg prednisolone on Day 4. The patient was then referred to our hospital for continuation of treatment, a close examination of her predisposition to collagen disease, and a close examination of the enlarged left axillary lymph node. No skin lesions or myositis were observed on physical examination, but a diagnosis of anti-MDA5 antibody-positive interstitial pneumonia was made as the blood test showed a high KL-6 level of 1060 U/ml (normal value <500 U/ml) and tested positive for anti-MDA5 antibodies. Treatment of anti-MDA5 antibody-positive interstitial pneumonia included the above-mentioned steroid treatment and initiation of tacrolimus (4 mg/day), followed by cyclophosphamide pulse (750 mg/day) and PE. Treatment resulted in mild improvement of the bilateral dorsal frosted shadows (**Fig. 1B**). The main clinical course after the start of treatment is shown in **Fig. 2**.

**Fig. 3:**
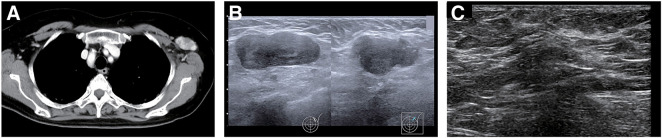
Imaging findings demonstrating left axillary mass lesion. (**A**) Mass shadow with contrast effect in the left axilla (mediastinal condition). (**B**) Left axillary ultrasound findings (at the time of referral to our department). (**C**) Left axillary ultrasound findings (after completion of neoadjuvant chemotherapy).

**Fig. 4:**
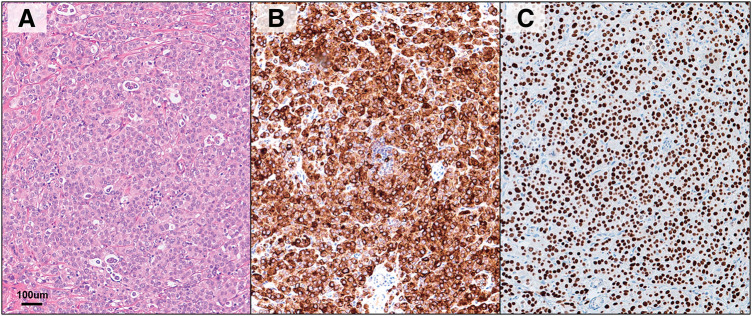
Histology of excised biopsy specimen. (**A**) Hematoxylin–eosin staining. (**B**) Anti-GCDFP-15 immunohistochemistry (**C**) Anti-GATA3 immunohistochemistry.

The patient was initially referred to the Department of Plastic Surgery for close examination of the enlarged left axillary lymph node. The chest CT scan findings prior to the start of treatment are shown in **Fig. 3A**. Although no lesions were detected within the mammary gland on ultrasound, 2 well-defined, coarse, hypoechoic masses of 35 and 19 mm in diameter, respectively, were found in the left axilla (**Fig. 3B**). Subsequently, an excisional biopsy was performed on the 35-mm large left axillary mass, and the pathological diagnosis was adenocarcinoma. The histopathological findings are shown in **Fig. 4**. Results of immunohistochemistry were as follows: GCDFP-15-positive, GATA3-positive, mammaglobin-negative, ER-negative, PgR-negative, HER2: score 1+, and a Ki67 labelling index of 40%. After the patient was referred to our department, further imaging tests, including mammography and contrast-enhanced breast magnetic resonance imaging, were conducted. However, neither revealed any lesions within the breast. The diagnosis of cTXN1M0 Stage IIA occult breast cancer (TN) was made on positron emission tomography-computed tomography (PET-CT) scan, which showed only hyperintense images in the left axilla. Upper and lower endoscopy performed at the same time to search for malignancy showed no abnormal findings.

**Fig. 5:**
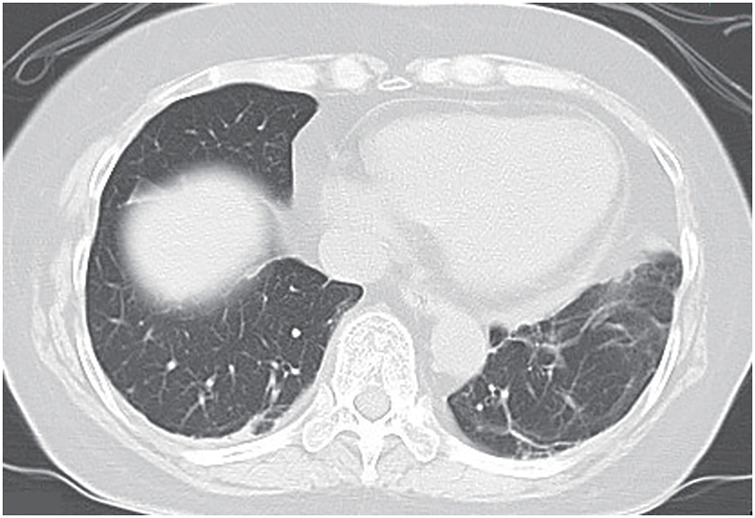
Chest CT scan during treatment for breast cancer. Chest CT scan findings during neoadjuvant chemotherapy for breast cancer are shown.

Chemotherapy is the recommended treatment strategy for TN breast cancer with lymph node metastases. The patient was immunosuppressed due to treatment for MDA5 antibody-positive interstitial pneumonia, but after consultation with a respiratory physician, the decision was made to use NAC, composed of 4 cycles of epirubicin and cyclophosphamide (EC) followed by weekly paclitaxel (12 weeks), to improve her breast cancer prognosis. During EC administration, granulocyte colony-stimulating factor (G-CSF) was prophylactically administered to help prevent febrile neutropenia (FN). The clinical response after NAC was a partial response. Ultrasound findings of the axillary lesion after completion of NAC are shown in **Fig. 3C**. There was no exacerbation of pneumonia during preoperative chemotherapy (**Fig. 5**), and the disease was well controlled. After completion of NAC, a left axillary lymph node dissection was performed as surgical treatment. The final pathological diagnosis showed metastases in 4 of the 10 lymph nodes removed, and a diagnosis of left latent breast cancer ypTXN2aM0 Stage IIIA was made. For occult breast cancer, guidelines recommend either mastectomy or, if mastectomy is not performed, radiation therapy.^8)^ However, the patient did not wish to undergo mastectomy. Meanwhile, postoperative radiotherapy was omitted because of concerns about exacerbating interstitial pneumonia and, instead, capecitabine, an oral fluoropyrimidine, was administered for 6 months. Subsequent follow-up has been conducted with ultrasound and mammography every 6 months. Three years after starting treatment for breast cancer, no evidence of recurrence has been observed, including in the right breast.

## DISCUSSION

The proportion of breast cancer as a malignant tumor complicating dermatomyositis in Europe and the United States was reported to be 17.3% by Callen.^9)^ Dermatomyositis complicated by malignancy has been reported to be associated with a poorer prognosis. In a cohort study by Sigurgeirsson et al. involving 618 patients with dermatomyositis or polymyositis, 32% developed malignancies within 3 years of diagnosis, and the majority of deaths were attributable to cancer.^10)^ Similarly, in a retrospective study by Liu et al. including 239 patients with dermatomyositis, 43 patients (18%) had coexisting malignancies, and their mortality rate was 42%, significantly higher than that of patients without malignancy (15%).^11)^ These findings suggest that the presence of malignancy in dermatomyositis is a major determinant of a poorer prognosis. Furthermore, some cases in the Liu et al. study showed improvement in dermatomyositis symptoms following successful cancer treatment, suggesting that malignancy may influence disease activity or treatment responsiveness. However, whether dermatomyositis affects cancer progression or interferes with its treatment remains unclear, and further investigation is needed to clarify this potential bidirectional relationship.

Interestingly, malignancy is reported to be less frequently associated with anti-MDA5 antibody-positive dermatomyositis compared to other forms of dermatomyositis.^12)^ In a cohort study by Fiorentino et al., only 1 out of 52 patients (2%) with MDA5 antibody positivity had malignancy.^2)^ In contrast, the incidence of malignancy associated with conventional dermatomyositis has been reported to be 20%–32% in large-scale cohort studies.^13,14)^ Thus, although the reason remains unclear, the frequency of malignancy in anti-MDA antibody-positive dermatomyositis is considered relatively low. Therefore, the current case was regarded as relatively rare.

The fatality rate of anti-MDA antibody-positive dermatomyositis combined with rapidly progressive interstitial pneumonia is very high and requires therapeutic intervention as soon as possible after diagnosis. Effective treatment methods include high-dose steroid therapy, calcineurin inhibitors, such as cyclosporine and tacrolimus, IVCY, and PE.^15,16)^ The initial treatment for pneumonia was effective in this case, allowing the patient to proceed with breast cancer therapy. However, there were risks associated with preoperative chemotherapy in an immunosuppressed state and the potential worsening of pneumonia due to cancer treatment. To mitigate these risks, careful monitoring was conducted throughout the treatment, and close collaboration with respiratory physicians was essential. As far as our search revealed, there have been very few reports on administering chemotherapy for breast cancer in patients with immunosuppression due to treatment for concurrent interstitial pneumonia. Koike et al. reported a case in which a patient successfully completed 4 courses of EC therapy for breast cancer while receiving 20 mg of methylprednisolone for the treatment of interstitial pneumonia.^17)^ On the other hand, previous studies have clearly demonstrated that the risk of FN is significantly higher in patients receiving corticosteroids.^18)^ For patients at high risk of developing FN, prophylactic administration of G-CSF and antibiotics is recommended.^19)^ The EC regimen used in this case has been reported to carry a risk of FN exceeding 20%,^20)^ indicating that the risk of FN was inherently high. Given that prophylactic administration of G-CSF is recommended for patients at high risk of FN,^19)^ G-CSF was administered as a preventive measure in this case. As a result, the patient was able to complete chemotherapy without developing FN. To establish guidelines for safely administering chemotherapy for breast cancer in an immunosuppressed state, further accumulation of cases is necessary.

Occult breast cancer is reported to account for 0.1%–0.3% of breast cancers.^21)^ Although there is still no clear treatment strategy for occult breast cancer, Japanese guidelines for the treatment of occult breast cancer recommend treatment of axillary lymph node metastases with an unknown primary site, based on histopathological findings on biopsy, in addition to local treatment (https://jbcs.xsrv.jp/guideline/2022/y_index/bq4/). As the subtype in this case was TN breast cancer, chemotherapy was recommended. Although no lesions were found in the breast, it is likely that an aggressive excisional biopsy, instead of assuming lymph node enlargement due to collagen disease, enabled the correct diagnosis and treatment to be carried out.

## CONCLUSIONS

In conclusion, we report a rare case of occult breast cancer in which a patient with anti-MDA5 antibody-positive associated interstitial pneumonia was successfully treated with preoperative chemotherapy and underwent curative surgery. Close collaboration with respiratory physicians was essential to ensure the smooth implementation of breast cancer treatment under the control of interstitial pneumonia.

## ACKNOWLEDGMENTS

The authors thank Clear Science Pty Ltd for language editing.

## DECLARATIONS

### Funding

The authors declare that they received no financial support pertaining to this report.

### Authors’ contributions

MI, YK, KA, AU, TK, SA, and TI treated this patient and provided clinical information.

ES conducted the pathological assessment.

MI and YH wrote the manuscript.

HK, KY, SA, and TI critically reviewed and edited the manuscript.

All authors contributed to discussions and agreed on the final version of the submitted manuscript.

### Availability of data and materials

Not applicable.

### Ethics approval and consent to participate

This work does not require ethical considerations or approval. Informed consent to participate in this study was obtained from the patient.

### Consent for publication

Written informed consent was obtained from the patient for the publication of this case report.

### Competing interests

The authors declare that they have no competing interests in this case.
